# Construction and validation of a psychometric scale to measure awareness on consumption of irradiated foods

**DOI:** 10.1371/journal.pone.0189314

**Published:** 2017-12-08

**Authors:** Tiago Rusin, Wilma Maria Coelho Araújo, Cristiane Faiad, Helio de Carvalho Vital

**Affiliations:** 1 College of Health Sciences, University of Brasília, Campus Darcy Ribeiro, Brasília, Brazil; 2 Ministry of the Environment, Esplanada dos Ministérios, Brasília, Brazil; 3 Department of Social and Work Psychology, Institute of Psychology, University of Brasília, Campus Darcy Ribeiro, Brasília, Brazil; 4 Department of Nuclear Engineering, Military Engineering Institute (IME), Rio de Janeiro, Brazil; University of Campinas, BRAZIL

## Abstract

Although food irradiation has been used to ensure food safety, most consumers are unaware of the basic concepts of irradiation, misinterpreting information and demonstrating a negative attitude toward food items treated with ionizing radiation. This research is aimed at developing a tool to assess the awareness on the consumption of irradiated food. The sample was composed by employees from different social classes and school levels of Brazilian universities, who reflect the end-users of the irradiated foods, representative of the views of lay consumers. The total number of respondents was 614. In order to assess the Awareness Scale on Consumption of Irradiated Foods (ASCIF), an instrument has been developed and submitted to semantic tests and judge’s validation. The instrument, that included 32 items, contemplated four construct factors: concepts (6 items), awareness (10 items), labeling (7 items) and safety of Irradiated foods (9 items). The data were collected by electronic means, through the site <https://pt.surveymonkey.com/>. By using exploratory factorial analysis (EFA) 4 factors have been found. They summarize the 31 items included. These factors account for 64.32% of the variance of the items and the internal consistency of the factors has been deemed good. An Exploratory Structural Equation Modeling (ESEM) was conducted to evaluate the factor structure of the instrument. The proposed instrument has been found to meet consistency criteria as an efficient tool for indicating assessing potential challenges and opportunities for the irradiated food markets.

## Introduction

Almost 800 million people (11 percent of the world’s population) are chronically hungry and 2 billion suffer from deficiency of micronutrients. Under a ‘business-as-usual’ scenario, without additional efforts to promote pro-poor development, some 653 million people would still be undernourished in 2030 [1]. An estimated 600 million–almost 1 out of 10 people–in the world become ill after consuming contaminated food and 420,000 die every year [2]. Among the different food preservation processes, food irradiation stands out for its main function being ensuring food safety [3].

According to Farkas & Mohácsi-Farkas [3] and Diehl [4], irradiated food is all food that has been intentionally subjected to the process of irradiation by means of ionizing radiation, whereas food irradiation is the term used to describe the process in which food is exposed to ionizing radiation such as gamma photons emitted by ^60^Co radioisotope (or very rarely ^137^Cs), X-rays generated by machines with a maximum energy of 5 MeV or accelerated electrons with a maximum energy of 10 MeV (kinetic energy).

Food irradiation has been mostly used for the following purposes: inhibition of budding; delay in maturation; reduction of microbial load; elimination of pathogenic microorganisms; sterilization and disinfection of grains, cereals, fruits and spices. Effects of irradiation depend on the type of food being treated and on the radiation dose being applied [5].

Despite the benefits of food irradiation, the acceptance of irradiated foods by consumers is still a major challenge [4, 6, 7, 8]. Researches have shown that the great barrier to the consumption of these products is the lack of knowledge or misconceptions by the population and professionals on the safety of irradiation and irradiated products [7, 8, 9, 10, 11, 12, 13, 14, 15].

The acceptance of new technologies of food production and processing by consumers is directly related to the credibility and trust in their sources of information. In a qualitative study conducted in 2006 [14] it was found that most of the Brazilian participants stated that they had never heard about food irradiation. The term irradiation often evokes a negative perception initially, since it encompasses the frightening word “radiation” that frequently produces unfounded associations, including the misleading perception of uncontrollability [15]. However, when properly informed about its real risks and benefits, most consumers will react positively to the acceptance of these foods [16, 17] and that supports the findings of [18] and [19].

It has been assumed that the population may be consuming irradiated food unawarely due to factors such as lack of knowledge on food irradiation, inappropriate information presented on food labels, and doubts about the safety of irradiated foods [6, 9, 12, 20, 21, 22, 23, 24].

Although many studies have already been performed, there is still lack of good instruments to evaluate the awareness of the consumption of irradiated foods, since most of the constructs used to assess social behavior in relation to irradiated foods did not undergo the rigorous methodology of construction and validation. Thus, it is necessary to build a valid instrument, robust and adapted to the local culture, which aims to evaluate the consciousness of the consumption of irradiated foods. In this context, the objective of this research was to develop a research tool to evaluate consumer knowledge about irradiated foods.

## Material and methods

### Study design

#### Literature review

The main objective of this stage was to identify studies carried out on the subject, as well as the existence of tools available for this analysis. Thus, the initial stage consisted in a literature search through PUBMED, SCOPUS, SCIENCE DIRECT, WEB OF SCIENCE, and INIS using the uniterms "food irradiation" AND (knowledge OR attitude OR perception OR awareness) AND consumer AND (survey OR questionnaire OR interview), without limitation of time and language. For the theoretical framework, classic publications of the area under study and gray literature data using Google Scholar and Proquest were still considered.

#### Construction of the instrument

This instrument called Awareness Scale for Consumption of Irradiated Foods (ASCIF) was constructed as a questionnaire and included the following factors: concepts, awareness, labeling and safety of irradiated foods. The proposed instrument measures the changes in positioning of the system of awareness regarding the consumption of irradiated foods.

The following constitutive definitions were adopted for the construct: Concepts (C), which evaluates the basic definitions, characteristics and principles of irradiated foods, food irradiation, processing, irradiation sources, ionizing radiation, absorbed dose, irradiators; Awareness (A), which evaluates the level of awareness on consumption of irradiated foods or ingredients. Also evaluated were data concerning quality and willingness to consume, characteristics that determine preference for acquisition and consumption of irradiated foods; Labeling (L), which assesses the knowledge of the legislation and the Radura symbol, the observation of irradiated food labels, the characteristics and information of foods bearing the indication of irradiated food on the label; Safety of Irradiated Foods (S), which assesses concern about the nutritional, chemical, physical, microbiological and nuclear safety of irradiated foods.

#### Semantic analysis

This analysis aimed to verify the comprehension of the items by members of the target population. The interviews were conducted individually or in groups of no more than three participants. The items were read aloud by the examiner and each participant was asked to judge them based on their intelligibility.

The semantic analysis of the instrument was performed with 20 participants aged between 18 and 70 years old, from different social classes and levels of schooling, that had been selected for convenience. Such inclusion criteria were aimed at avoiding bias and reaching a broad and lay public on the subject of irradiated foods. The Informed Consent Form (ICF) was dated and signed by the participants.

#### Judge analysis

Based on the criteria for the definition of judges used by Medeiros et al. [25], the instrument was submitted to semantic analysis and validation by five food professionals distributed as follows: government representatives (n = 2), university professors (n = 2) and food consultant (n = 1). They received the evaluation form for the proposed items together with ICF and returned this document, dated and signed.

In order to conduct the analysis of judges, a form containing the operational definition of the construct, the proposed items and the instructions for its analysis was prepared. The evaluation of the items included the adequacy of the item to the factor and the clarity. The form also included space for judges to make suggestions.

Data were analyzed considering each of the evaluated items and suggestions presented for reformulation of items. The criterion for maintenance of the item in the scale was to obtain at least 80% agreement between judges in each of the requirements [26]. The criterion for eliminating the item was marking the item as incomprehensible by at least one of the judges or; when the item did not obtain at least 80% agreement between the judges.

### Sample

The sample was composed by employees from different social classes and school levels, identified in the lists of servers of the Secretariats of Human Resources of Brazilian universities of the five regions of the country, giving national representation to the sample. These employees reflect the end-users of the irradiated foods, representative of the views of lay consumers. The following universities were selected: University of Brasília, Rio de Janeiro State University, Fluminense Federal University, University of Sao Paulo, Federal University of Pará, Rural Federal University of Pernambuco, Federal University of Pernambuco, Goias Federal University, Federal University of Minas Gerais, Federal University of Southern Frontier, Federal University of Parana and Federal University of Rio Grande do Sul. Respondents from all school levels (excluding the illiterate because the questionnaire requires their reading and comprehension) were considered for the research, with ages ranging from 18 to 70 years of age, of both sexes and of different social classes, with social variability being desirable in the sample. To these groups, the instrument was applied online and the total number of respondents was 624.

### Data collect

The data were collected by electronic means, through the site <https://pt.surveymonkey.com/>. In order to evaluate the knowledge and behavior of consumers as well as about the knowledge and habits of consumers on irradiated foods, a questionnaire with a Likert scale of agreement was used. The scale offered five options of answer for each affirmative, ranging from strongly disagree (1), disagree (2), neither agree nor disagree (3), agree (4), and strongly agree (5). This instrument consisted of items that identified the socio demographic characteristics of the sample, behavior measures and measures of knowledge about the subject.

### Statistical treatment

The data gathered were analyzed and validated by means of statistical treatments. The analysis and validation of the data provided the results and solution strategies for the problems identified.

Evidence of validity was verified by using exploratory factorial analysis (EFA) in order to evaluate the psychometric quality of the instrument. The reliability of the instrument was evaluated based on the internal consistency, obtained through the Cronbach´s Alpha coefficient. These statistical analyses were performed using IBM SPSS (version 21). An Exploratory Structural Equation Modeling (ESEM) was conducted to evaluate the factor structure and the StdYX coefficients were calculated using the MPlus software.

This research project was approved by the Research Ethics Committee/ Faculty of Health Sciences/ University of Brasília on August 08, 2016 (Certificate of Presentation for Ethical Appreciation 57419216.2.0000.0030).

## Results

### Demographics

Of the total sample (n = 624), 10 respondents did not complete the entire questionnaire and their answers were not included in the analyzes (n = 614, response rate = 98,4%) (see S1 File on Supporting information). The majority of respondents were woman (57%) between the ages of 30 and 39 years (33.2%), with a doctorate (PhD) degree (57.3%) and with an average family income of 10 to 20 minimum wages–equivalent to 3.000 to 6.000 dollars per month (46.6%). In addition, 72.3% had a companion, 82.2% were public-sector employees, 97.1% were native Brazilian, with 2 to 5 people living in the same house (44.3%), 82.4% were responsible for grocery shopping in home and 77.7% were from Southeastern Brazil (Table 1).

**Table 1:** Frequency of selected demographic variables of respondents (n = 614).

Demographic variables	Number of participants (%)
**Gender**:	
Male	264 (43.0)
Female	350 (57.0)
**Age (years)**:	
< 20	2 (0.3)
20–29	80 (13.0)
30–39	204 (33.2)
40–49	138 (22.5)
> 50	190 (30.9)
**Educational level**:	
Elementary School	1 (0.2)
High school	13 (2.1)
Bachelors	57 (9.3)
Specialization	75 (12.2)
Masters	116 (18.9)
Doctorate degree	352 (57.3)
**Average monthly family income (in minimum wage—R$)**:	
< 2	6 (1.0)
2–5	45 (7.3)
5–10	146 (23.8)
10–20	285 (46.4)
> 20	132 (21.5)
**Marital status**:	
With partner	444 (72.3)
Without partner	170 (27.7)
**Profession**:	
Public-sector employees	505 (82.2)
Private-sector employees	60 (9.8)
Self Employed	7 (1.1)
Student	11 (1.8)
Intern	0 (0.0)
Retired	1 (0.2)
Informal	0 (0.0)
Military	10 (1.6)
Other	20 (3.3)
**Nationality**:	
Native Brazilian	596 (97.1)
Brazilian naturalized	10 (1.6)
Foreign	8 (1.3)
**How many people live in your house besides you?**	
I live alone	76 (12.4)
Up to 2 people	253 (41.2)
From2 to 5 people	272 (44.3)
More than 5 people	13 (2.1)
**Are you responsible for grocery shopping in your home?**	
Yes	506 (82.4)
No	108 (17.6)
**Region of residence (Brazil)**:	
North region	3 (0.5)
Northeast region	11 (1.8)
Midwest region	477 (77.7)
Southeast region	104 (16.9)
South region	19 (3.1)

### Construction of ASCIF

The original version of the Awareness Scale for Consumption of Irradiated Foods (ASCIF) contained 92 items that were elaborated based on validated technical documents [6, 20, 22, 23], periodicals [3, 4, 7, 8, 12, 13, 21, 24], and related literature [27]. Four factors were theoretically identified: concepts (C; 19 items), awareness (A; 20 items), labeling (L; 24 items) and safety of irradiated foods (S; 29 items).

In the semantic analysis carried out with 18 employees and 4 interns/students from different undergraduate courses at the University of Brasilia, items with ambiguous meaning and the presence of words of difficult comprehension were detected for a public lay on the subject, mostly unfamiliar with the technical terms. Thus, from the 92 items initially proposed, 63 items remained unchanged and 29 items were eliminated. The eliminated items were related to: concepts (7 items), awareness (5 items), labeling (10 items) and safety of irradiated foods (7 items).

Based on criteria for maintaining or eliminating items determined by the judges, most of the remaining items (32 of the 63 items, a total of 50.8%) were kept in the instrument due to their relevance. The 31 eliminated items were distributed as follows: concepts (6 items), awareness (5 items), labeling (7 items) and safety of irradiated foods (13 items). Thus, the pilot version of the scale presented a total of 32 items: 6 items were related to concepts, 10 items to awareness, 7 items to labeling and 9 items to safety of irradiated foods.

### Exploratory analysis of data

#### Analysis of assumptions

Initially an exploratory analysis was performed in order to better understand the characteristics of the data, as well as to identify possible missing data and extreme cases (outliers). The descriptive statistics of the collected data were performed. Based on them, it was concluded that there were 10 participants with missing data on variables and they were excluded from the analysis. Nineteen univariate outliers for the variables were found and used to analyze assumptions. A total of 33 multivariate outliers were found by using Mahalanobis distance technique, considering p<0.001 and influential observations according to the Leverage measure. Cook′s distance method did not yield any event greater than 1, indicating that the outlier did not substantially influence the results. Considering that the exploratory factorial analysis is robust and these cases would not influence the results, it was decided to maintain multivariate outliers identified by the Leverage measure and Mahalanobis distance, and that resulted a in a total of 614 cases for the analyzes.

Statistical values (histograms, skewness and kurtosis values) and significance tests (Shapiro-Wilk) indicated normal distribution. Considering that the assumption of univariate normality was met, it is assumed that multivariate normality was also met. Although univariate normality does not guarantee multivariate normality, if all variables meet this condition, then deviations from multivariate normality are innocuous [28].

The assumption of linearity was analyzed from inspecting residues. It was verified that the points were randomly distributed around zero, so that the linearity assumption could be assumed.

The assumption of singularity was analyzed through VIF and tolerance. It was observed that all variables met the criterion of VIF less than 5 and a tolerance greater than 0.1 so that all variables met the assumption of uniqueness, according to [28].

#### Exploratory factorial analysis

Exploratory factorial analysis was initially used to test the assumptions made. Factorization of the correlation matrix was evaluated through inspection of the correlation matrix; verification of the matrix determinant; and Kaiser-Meyer-Olkin (KMO) sample adequacy index. The correlation matrix pinpoints correlations among variables. The determinant of the matrix has a low and nonzero value; The KMO of 0.952, is considered marvelous by [29] classification, and Bartlett′s sphericity test significantly indicated matrix factorability. The principal component analysis was performed and through no extreme values (0–1) were found, indicating that there were no problems in the matrix.

The total variance explained shows that using the eigenvalues criterion greater than 1, up to five of the components would be factorizable. The scree plot corroborates the analysis of the total variance explained by demonstrating up to five factors, based on the eigenvalue above 1. The component matrix was generated to visualize the contribution of the variance of each variable in each component. It was observed that the first four components added more information, conform the principle of parsimony.

To confirm the number of factors to be extracted, the parallel analysis was performed comparing the empirical matrix with a random matrix. To identify the value of the random eigenvalues, the RanEigen software [30] was used. By comparing the empirical matrix data with the random matrix, it was found that 4 factors to be extracted. Considering all possible criteria for choosing the number of factors to be extracted, it was decided up to 4 factors would be extracted, since most of the analyzes indicated that this would be the best option.

Considering the results obtained in the analysis of main components (PC), the extraction and rotation of the main factors (PAF) was performed. PROMAX rotation was used because the factors were intercorrelated, indicating that the factors were oblique. Table 2 presents the scheme for extraction of 4 factors, and shows the correlation between factors 1, 2 and 4; 2, 1 and 4; and 4, 1 and 2, using coefficients higher than 0.3 as criterion.

**Table 2:** Matrix of factor correlations.

**Factor**	**1. S**	**2. C**	**3. L**	**4. A**
**1. S**	1.00			
**2. C**	0.62	1.00		
**3. L**	-0.26	0.06	1.00	
**4. A**	0.51	0.49	-0.04	1.00

S = Safety of irradiated foods; C = Concepts; L = Labeling; A = Awareness.

Extraction Method: Main Axis Factor.

Rotation method: Promax with Kaiser normalization.

All presented correlations are significant (p<0.05).

Table 3 shows the extraction of 4 factors with corresponding information. Item Q17 was eliminated because it did not yield a factorial load greater than 0.4 for the extracted factors.

**Table 3:** Exploratory factor analysis of the ASCIF using principal components factoring with promax rotation.

Items	Mean (s.e.)	Factor loadings^a^			
		1^b^	2^c^	3^d^	4^e^
Q30. I consider that irradiated foods are not harmful to health in the long term.	3.01 (0.04)	0.93			
Q11. I would encourage consumption of irradiated foods.	2.84 (0.04)	0.92			
Q29. I consider that irradiated foods are not harmful to health in the medium term.	3.12 (0.04)	0.92			
Q31. I consider that irradiated foods are not harmful to the health of future generations.	3.03 (0.05)	0.92			
Q13. I would consume irradiated food because I know that these are safe for consumption.	3.11 (0.05)	0.86			
Q12. I would consume irradiated foods, as I know they do not cause health damage.	3.10 (0.05)	0.86			
Q28. I consider that irradiated foods are not harmful to health in the short term.	3.24(0.04)	0.84			
Q15. I approve the consumption of irradiated foods.	3.16 (0.05)	0.80			
Q27. I feel safe about the consumption of irradiated foods.	3.01 (0.05)	0.75			
Q10. I would be willing to pay more for irradiated food.	2.38 (0.04)	0.70			
Q9. I would consume irradiated food.	3.35 (0.05)	0.70			
Q22. I have confidence in buying a food when I read on the label the following information "food treated by irradiation process".	3.07 (0.05)	0.69			
Q24. I would buy irradiated food because I know this process does not make the food radioactive.	3.48 (0.05)	0.63			
Q26. Irradiated foods are nutritional safe.	3.30 (0.04)	0.61			
Q32. The World Health Organization (WHO) and the United Nations (FAO) recommend the irradiation of food.	3.11 (0.03)	0.59			
Q2. Food irradiation can be used to reduce microbial load on food.	4.07 (0.04)		0.83		
Q7. Food irradiation can be used to increase shelf life.	3.98 (0.04)		0.72		
Q3. The irradiation of food can be used to inhibit the budding of bulbs, roots and tubers.	3.58 (0.04)		0.61		
Q5. The minimum absorbed dose by the irradiated food must be sufficient to achieve the intended purpose.	3.77 (0.04)		0.57		
Q6. Brazil authorizes the use of food irradiation.	3.63 (0.03)		0.56		
Q25. Irradiated food is microbiologically safe.	3.50 (0.04)		0.51		
Q4. Food irradiation can be used to delay the ripening of fruits.	3.57 (0.04)		0.47		
Q1. Irradiated food is different from radioactive food.	3.99 (0.05)		0.45		
Q19. All foods that undergo irradiation should have this information highlighted on the product label.	4.53 (0.03)			0.90	
Q21. I consider the symbol of Radura important in the labels of irradiated foods.	4.45 (0.03)			0.85	
Q23. The food label should highlight the information of irradiated food.	4.40 (0.03)			0.80	
Q20. I consider that the additional information contained in the labels of irradiated foods is important.	4.45 (0.03)			0.78	
Q16. I consider it necessary to carry out educational campaigns to inform the population about the irradiation of food.	4.59 (0.03)			0.60	
Q14. I know some irradiated food.	2.58 (0.06)				0.70
Q18. I know Radura, the symbol used to represent irradiated food.	2.45 (0.06)				0.60
Q8. I consciously consume irradiated food.	2.40 (0.05)				0.58
Eigenvalues		13.42	3.88	1.87	1.42
Variance accounted for (%)		41.92	12.12	5.84	4.43

^a^ Loadings < 0.40 are omitted.

^b^ Safety of irradiated foods (S).

^c^ Concepts (C).

^d^ Labeling (L).

^e^ Awareness (A).

#### Reliability

To test the internal consistency of the factors, Cronbach alpha index was used. The number of items for Factors 1, 2, 3 and 4, were respectively: 15, 8, 5 and 3 while the corresponding alpha indices were: 0.97, 0.84, 0.89 and 0.80. After these analyzes 4 factorial scores were created based on the simple mean, in order to facilitate future analyzes. Factor 1 includes "Safety of irradiated food (S)", factor 2 "Concepts (C)", factor 3 "Labeling (L)" and factor 4 "Awareness (A)", according to Table 3.

#### Exploratory Structural Equation Modeling (ESEM)

**Fig 1:**
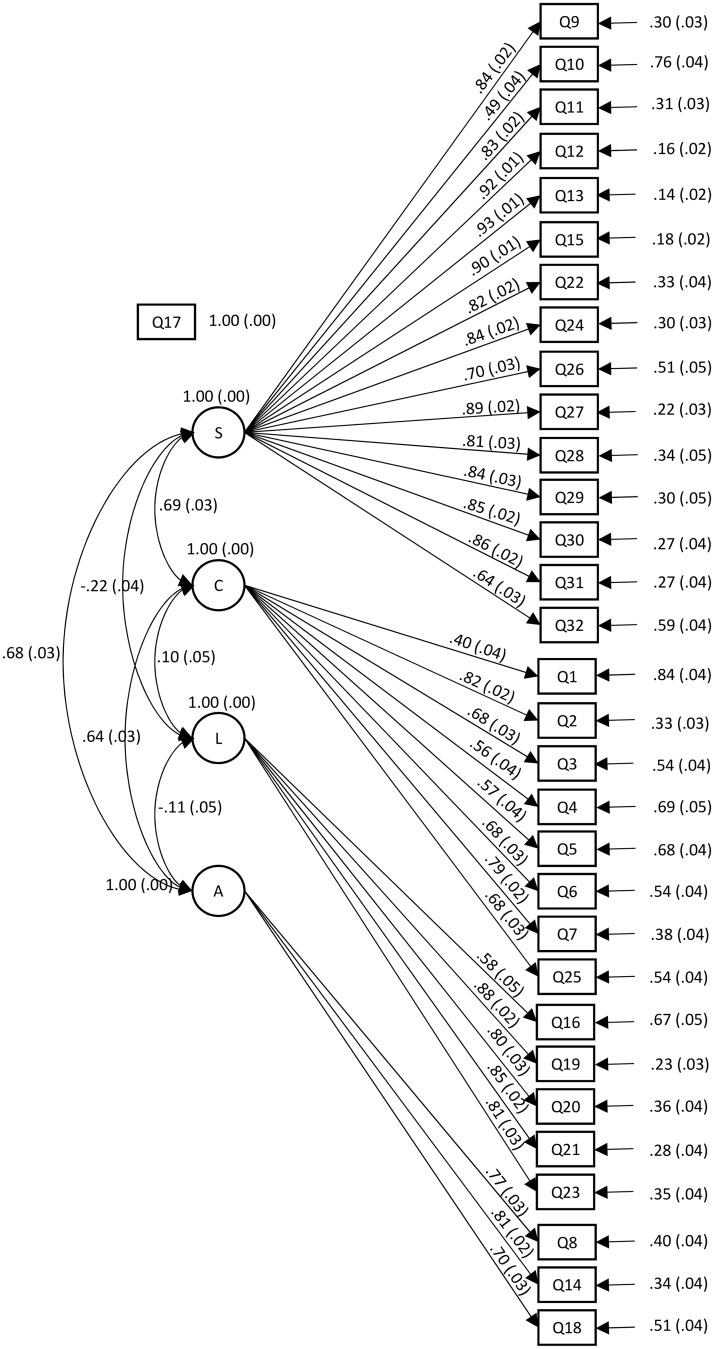
ESEM with the StdYX coefficients and standard errors (p<0.05).

For analysis of evidence of instrument validity, based on the results from the EFA and on the reliability of the instrument, the Exploratory Structural Equation Modeling (ESEM) was also calculated. The factor structure adequacy was evaluated by using the comparative fit index (CFI), root mean square error of approximation (RMSEA) and the standardized root mean square residual (SRMR). Fig 1 presents the structural model with the StdYX coefficients and their standard error, all presented coefficients are significant (p<0.05).

The fit index was CFI = 0.855; RMSEA (90% CI) = 0.077 (0.074–0.080); SRMR = 0.055. For Factor 1 "Safety of irradiated foods (S)" the StdYX coefficients varied between 0.49 (0.04) and Q10 and 0.93 (0.01) for Q13. For Factor 2 "Concepts (C)" StdYX ranged from 0.40 (0.04) to Q1 to 0.82 (0.02) for Q2. For Factor 3 "Labeling (L)" the coefficients varied between 0.58 (0.05) and Q16 and 0.88 (0.02) for Q19. While for Factor 4 "Awareness (A)" the StdYX coefficients ranged from 0.70 (0.03) to 0.81 (0.03). Further details are shown in Fig 1.

## Discussion

### Evidences of validity

The purpose of this research was to construct and validate a measurement instrument aimed at evaluating the consciousness regarding the consumption of irradiated foods. It is hoped that the proposed instrument will be used by researchers in order to contribute scientifically to the development of new strategies of diffusion of knowledge on the applications of food irradiation and its attractive potential to ensure safety of food products.

According to [31], validity is an integrated evaluative judgment of the degree to which empirical evidence and theoretical justifications support the adequacy of inferences and actions based on test results or other modes of evaluation. According to the [32], validity refers to the degree to which evidence and theories support interpretations of test scores for certain uses proposed for it. The validation process involves the accumulation of relevant evidence to provide a sound scientific basis for interpretations of the proposed scores.

ASCIF items were based on valid, periodical, and related literature on food irradiation, presenting evidence of content validity in accordance with the requirements of the [32].

The semantic and judges′ analysis contributed substantially to the process of constructing the new scale, indicating that they are relevant methodological procedures that can be used to construct new measurement instruments, indicating other validity evidences such as face validity, of a subjective nature.

According to the agreed rule, the sample met the requirements of a minimum number of 200 subjects and at least 5 participants per variable, so the criterion of [33] was satisfied with the current data base (614 individuals and 32 items). To avoid errors of record, all the values imputed to the items were within the minimum and maximum considered. The assumptions of normality, linearity and singularity were met in accordance with the precepts of [34].

In order to decide on the factor of the matrix, four parameters can be made available from analysis of the R matrix, namely: the size of the intercorrelations, the AIC, the KMO and the square of the multiple correlations (R^2^). The obtained KMO indicates that the image/anti-image relation is considered excellent, so the R matrix can be factorized according to such criterion. It is known that the further away from 1 is such factor, the more questionable becomes the statistics, because such an event indicates that the variables do not have much in common. Kaiser [29] characterizes the KMO values as follows: 0.90 marvelous; 0,80 meritorious; 0.70 median; 0.60 mediocre (modest); 0.50 miserable and; below 0.50 unacceptable [35]. The KMO value found in this research (0.952) is marvelous and therefore allows factor analysis.

In order to decide the number of factors the R matrix comprises, the analysis of the main components PC was used. The use of PC in the initial stages of the analysis is justified by the fact that it uses all the variance of the variables and not only the covariance. In addition, it uses eigenvalues and their representation in the scree plot as criteria for the number of factors, and is based on an analysis of an R matrix adjusted with 1 on the diagonal [28, 35].

The interpretation of the eigenvalues of the empirical matrix legitimates the extraction of up to 5 components of the matrix R, according to the criterion of K1. The criterion K1 is very lenient, suggesting the existence of a number of components greater than in fact exist in the matrix [28]. Such issue still requires further investigation. Therefore, it was more enlightening to verify the same information expressed by the scree plot (graphical interpretation), and especially the information given by the random eigenvalues that were verified in this research by means of a random matrix. There are four empirical eigenvalues that surpass the largest random eigenvalue. Thus, the extraction of up to four factors from the empirical matrix is justified because it is believed that they contain the greatest amount of true information produced in this research.

Interpreting the variance of the R matrix, we lead to the conclusion that it was good, because about 35.68% of the variance is unexplored. This unexplored variance of the variables is distributed into three possible categories: error variance, specific variance, and non-extracted common variance. Of these, the common non-extracted variance is of interest for the factorial analysis, since its presence indicates that the number of factors extracted was not enough to explain the covariance between the variables and, thus, the factorial analysis is no longer adequate. This part of the variance of interest is expressed by the covariance still found in the residual matrix, which here was very small. In this way, the extraction of 4 factors seemed to be the most adequate for the R matrix found.

Factor analysis indicated that the 31 items included can be summarized in 4 factors. The first factor grouped 15 items related to safety of irradiated foods. The second factor grouped 8 items related to concepts (C). The third factor grouped 5 items related to labeling (L). Finally, the fourth factor grouped 3 items related to awareness (A).

There was a high correlation between factors 1 and 2 (0.62); 1 and 4 (0.51); 2 and 4 (0.49) and low correlations of factors 1, 2 and 4 with factor 3 (-0.26; 0.06; -0.04). It is understood that the safety of irradiated foods, concepts and awareness are correlated and labeling is a subject not correlated with the others, in this way the theoretical model adopted allowed this degree of association between the factors.

The correlations found are in agreement with [18], since conventional consumer attitudes towards food irradiation can be positively influenced by educational efforts as the general public is still mostly unaware of its benefits and advantages [15]. Therefore, consumers need information on protective technologies, such as food irradiation [36].

The low correlation found for the labeling (factor 3) possibly results from the scarce knowledge on the process and its symbol (Radura). According to [21], very few respondents (95.8%) were acquainted with the Radura symbol, supporting the findings of this work.

Labeling of irradiated foods is of paramount importance for consumers to meet their expectations and preferences during purchases. However, there is a great gap in the identification of such foods and the symbol of Radura is often of little help, since it is associated with food irradiation by relatively few consumers. Nevertheless, most respondents saw labeling as an information issue necessary to ensure consumer choice [37]. According to [38], the optimal package for irradiated strawberries should bear the following information, according to the Ratings Based Conjoint Analysis (RBCA) and Modified-Choice Based Conjoint Analysis (MCBCA) results: “Food treated by ionization process” or “Food treated by irradiation process”, “To ensure freshness and quality for a longer time” and the presence of the Radura symbol.

The interpretation of the internal consistency analysis showed that the factors presented with good indices (Cronbach’s alpha), since values higher than 0.80 were found for all four factors. As the data reveal the consistency or invariance of the factors, this analysis shows that the four factors are guaranteed to appear in other studies because their items are related to each other and seem to represent a good construct.

The Exploratory Structural Equation Modeling (ESEM) gives access to all the usual Structural Equation Modeling (SEM) parameters and the loading rotation gives a transformation of structural coefficients as well. Standard errors and overall tests of model fit are obtained [39].

The ESEM of the ASCIF instrument presented good StdYX coefficients comparable to the EFA findings. The coefficients between the factors, very similar to the correlation matrix (Table 2), are important findings. Exploratory Structural Equation Modeling can serve as a preparation for future research to perform confirmatory factor analysis and Structural Equation Modeling (SEM) of the instrument.

### Limitations

The convenience sample used for validation of the ASCIF instrument included the majority of postgraduate respondents (masters and doctors), which does not reflect the Brazilian population profile, so the generalization of our results is limited, however it can be said that it reflects the profile of the final consumer of irradiated food in the country, as they demonstrate a better knowledge on food irradiation. Additional studies with different population profiles, social and cultural classes should be performed to confirm the generalization of our results. It is also suggested that the ASCIF instrument be adapted to other languages and cultures in order to be applied in other countries, allowing the comparison of the results.

## Conclusion

This research sought to fill the gap of validated instruments to evaluate the consciousness regarding the consumption of irradiated foods. It was possible to construct a psychometric scale to measure the awareness scale for the consumption of irradiated foods, that exhibits good indications of validity. Four factors were found: concepts, awareness, labeling and safety of irradiated foods representing 31 items of the instrument, with good indices of internal reliability. The ASCIF instrument demonstrates potential to be adapted to other languages and cultures, being an unprecedented instrument to measure knowledge in the food domain, indicating potential challenges and opportunities for the commercialization of irradiated foods.

## Supporting information

S1 File Database used in the analyzes (n = 614). (SAV)
